# Real-world study of PD-1/L1 immune checkpoint inhibitors for advanced non-small cell lung cancer after resistance to EGFR-TKIs

**DOI:** 10.3389/fonc.2023.1217872

**Published:** 2023-07-18

**Authors:** Kunchen Wei, Chao Zhou, Yang Chen, Xiao Feng, Hao Tang

**Affiliations:** Department of Respiratory and Critical Care Medicine, Changzheng Hospital, Navy Medical University, Shanghai, China

**Keywords:** non-small cell lung cancer, epidermal growth factor receptor, immune checkpoint inhibitor, nomogram, EGFR TKI resistance

## Abstract

**Background:**

Programmed cell death-1 (PD-1) and its ligand 1 (PD-L1) inhibitors have achieved good efficacy and safety in patients with advanced EGFR mutation-negative non-small cell lung cancer (NSCLC), but their efficacy in patients with previous EGFR mutations is limited. The aim of the present study was to explore the efficacy of PD-1/L1 immune checkpoint inhibitors for the treatment of patients with advanced NSCLC who are resistant to EGFR-TKIs

**Methods:**

This retrospective study included 123 patients with stage IV NSCLC who received treatment in Shanghai Changzheng Hospital between January 2019 and January 2022 after failure of first-line EGFR-TKIs. Of them, 39 received ICIs + chemotherapy and anti-angiogenic drugs (ICIs+BCP group), 51 received ICIs monotherapy (ICIs group), and 33 received chemotherapy and anti-angiogenic drugs (BCP group). The gender, age, smoking history, ECOG score, EGFR mutation type, PD-L1 TPS expression, and the first routine blood index before second-line treatment of all enrolled patients were recorded, and their clinical outcomes and prognosis factors were analyzed.

**Results:**

There was no significant difference in the objective response rate (ORR) and disease control rate (DCR) between the three groups. Patients in ICIs+BCP group had better prognosis than those in ICIs monotherapy group (PFS:9.5 *vs.* 4.64 months, p<0.001; OS: 16.97 *vs.* 7.9 months p<0.001) or BCP group (9.5 *vs.* 6.48 months, p<0.005; OS: 16.97 *vs.* 11.39 months p<0.005).

**Conclusion:**

Our findings suggest that in the real-world practice in China, PD-1/L1 immune checkpoint inhibitors combined with chemotherapy and anti-angiogenic drugs are effective for the treatment of patients with advanced NSCLC who are resistant to EGFR-TKIs.

## Background

Epidermal growth factor receptor tyrosine kinase inhibitors (EGFR-TKIs) are the first-line standard of care for advanced non-small cell lung cancer (NSCLC) patients with EGFR-sensitive mutations (1, 2). Unfortunately, drug resistance often develops following EGFR-TKIs treatment and the mechanisms of resistance are variable (3). Currently, there are limited follow-up therapies for patients who are resistant to EGFR-TKIs. Programmed cell death 1 (PD-1) and its ligand 1 (PD-L1) inhibitors have achieved good efficacy and safety in some patients with advanced EGFR mutation-negative NSCLC, but their benefits in patients with previous EGFR mutations are limited (4–6). The aim of the present study was to investigate the efficacy of immune checkpoint inhibitors (ICIs) as the second line treatment for stage IV NSCLC patients following failure of first line EGFR-TKIs by retrospectively analyzing the clinicopathological features of patients with advanced NSCLC who were admitted to Changzheng Hospital (Shanghai, China) between January 2019 and January 2022, their progression survival (PFS), overall survival (OS), the objective response rate (ORR), disease control rate (DCR), and EGFR driver mutation.

## Patients and methods

### Patient selection

The medical records of patients who failed the treatment with first-line EGFR-TKIs were analyzed retrospectively, in whom histological or somatic cytological investigation and second-generation sequencing study were performed to determine the presence or absence of EGFR driver mutations. Patients who met the following criteria were included for further analysis: (1) age ≥ 18 years and ≤ 75 years; (2) with histologically, cytologically or pathologically confirmed stage IV NSCLC in accordance with the TNM criteria specified in the 2017 8^th^ Edition of the International Association for the Study of Lung Cancer (IASLC); (3) with at least one quantifiable lesion in accordance with RECIST 1.1 standards; (4) confirmation by next generation sequencing testing as having EGFR driver gene mutation possibly with another positive driver gene; (5) received first-line targeted therapy with first/second generation EGFR-TKIs, including gefitinib, erlotinib, afatinib, and dacomitinib; (6) disease progression after treatment with first-line EGFR-TKIs; and (7) second-generation sequencing test showing clear negativity for EGFR T790M again after resistance to first-line EGFR-TKIs. The main exclusion criteria were (1) genetic testing suggesting T790M positivity again after resistance to first-line EGFR-TKIs; (2) inability to proceed to second-line treatment due to severe toxic and adverse effects; and (3) pathologically confirmed small cell lung cancer after resistance to first-line EGFR-TKIs. This study was approved by the ethics committee of Shanghai Changzheng hospital (2021SL018). Because this was a retrospective cohort study, informed consent was waived.

### Study design

According to their second-line treatment modality, all study participants were given first-line EGFR-TKIs and then divided into three groups: ICIs combined with platinum-containing two-drug chemotherapy and anti-angiogenic drugs (ICIs+BCP group), ICIs monotherapy group (ICIs group), and platinum-containing two-drug chemotherapy combined with anti-angiogenic drugs (BCP group). Gender, age, smoking history, ECOG score, EGFR mutation type, PD-L1 tumor cell proportion score (TPS), first routine blood parameters before second-line treatment including neutrophil, lymphocyte, monocyte count and platelet counts, and serum inflammation-related factors were recorded in all patients. In addition, general information including the neutrophil-to-lymphocyte ratio (NLR), monocyte-to-lymphocyte ratio (MLR) and platelet-to-lymphocyte ratio were measured. All patients were followed up until January 2022, when their PFS, OS, ORR and DCR were calculated to determine the effectiveness of ICIs as the second-line treatment for patients with advanced NSCLC who were resistant to EGFR-TKIs. 20 NSCLC patients meeting inclusion criteria from February 1, 2022 to January 31, 2023 as an external validation set.

### Statistical analysis

This study was conducted using STATA (version 16.0), R (version 4.0.3), SPSS (version 26.0) and GraphPad Prism (version 8.0.1) software for statistical analysis and data visualization. Measurement data are expressed as the mean ± standard deviation (SD), and enumeration data are expressed as the percentage (%). Analysis of variance (ANOVA) was used for comparison between groups for measurement data, and χ2 test was used for comparison between groups for enumeration data. Kaplan-Meier method was used to assess OS and PFS between patient groups, and Log-rank method was used to analyze survival differences. Univariate and multifactorial COX regression analyses were used to screen for independent prognostic factors. R software and associated R package were used to construct Nomogram prediction models. The closer the AUC value to 1 indicates better discrimination. *P* < 0.05 was considered statistically significant.

## Results

### Patient characteristics

A total of 442 patients diagnosed with stage IV NSCLC were collected in this study, excluding 81 patients whose disease had not yet progressed after treatment with first-line EGFR-TKIs, and a total of 361 patients showed disease progression requiring second-line treatment, of whom 43 patients received targeted therapy with third-generation EGFR-TKIs, and 123 patients met the inclusion criteria of this study. Analysis of the general data of all enrolled patients revealed 123 patients with advanced NSCLC, who were classified as three groups: 39 in ICIs+BCP group, 51 in ICIs group, and 33 in BCP group. ANOVA analysis showed significant differences in age distribution, ECOG score, EGFR mutation type and PD-L1 TPS expression between the three groups (p < 0.05). The details are listed in **Table 1**.

**Table 1:** Patient characteristics (*N* = 123).

Characteristic	ICIs+BCP (*N*=39)	ICIs (*N*=51)	BCP (*N*=33)	*p*
Age (`x ± s)	64.2±11.9	63.5 ± 13.9	60.4 ± 10.8	<0.001
≦65	17 (43.6%)	40 (78.4%)	27 (81.8%)	
>65	22 (56.4%)	11 (21.6%)	6 (18.2%)	
Sex				
Male	24 (61.5%)	25 (49.0%)	15 (45.5%)	0.338
Female	15 (38.5%)	26 (51.0%)	18 (54.5%)	
Smoking history				
No	30 (76.9%)	33 (64.7%)	21 (63.6%)	0.373
Yes	9 (23.1%)	18 (35.3%)	12 (36.4%)	
ECOG score				
0	25 (64.1%)	44 (86.3%)	30 (90.9%)	0.017
1	13 (33.3%)	7 (13.7%)	3 (9.1%)	
2	2(2.6%)	0 (0.0%)	0 (0.0%)	
EGFR mutation				
19del	27 (69.2%)	40 (78.4%)	14 (42.4%)	0.003
21L858R	12 (30.8%)	11 (21.6%)	19 (57.6%)	
PD-L1 TPS				
<1%	27 (69.2%)	42 (82.4%)	18 (54.5%)	0.023
≧1%	12 (30.8%)	9 (17.6%)	15 (45.5%)	

ECOG, Eastern Cooperative Oncology Group; EGFR Epidermal, Growth Factor Receptor; TPS, Tumor cell Proportion Score.

### Therapeutic efficacy

**Figure 1:**
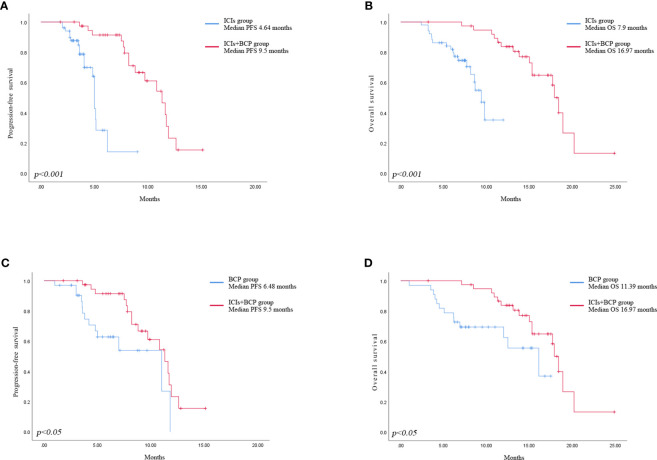
Kaplan-Meier analysis of progression-free survival in ICIs group and ICIs+BCP group **(A)**. Overall survival in ICIs group and ICIs+BCP group **(B)**. Progression-free survival in BCP group and ICIs+BCP group **(C)**. Overall survival in BCP group and ICIs+BCP group **(D)**.

Until January 2022, no patient achieved complete remission (CR) in all three groups. The number of patients who achieved partial remission (PR) was 6 (15.4%) in ICIs+BCP group, 10 (19.6%) in ICIs group, and 4 (12.1%) in BCP group. Stable disease (SD) in 30 (76.9%), 39 (76.5%) and 26 (78.8%) patients of the three groups respectively, 3 (7.7%), 2 (3.9%) and 3 (8.3%) patients demonstrated progressive disease (PD). There were no significant differences in ORR and DCR between the three groups (**Table 2**). Log-rank test of OS and PFS in 39 cases in ICIs+BCP group and 51 cases in ICIs-alone group showed that the overall prognosis in ICIs+BCP group was significantly better than that in ICIs-alone group [OS: 16.97 months (15.11-18.84 months) *vs.* 7.9 months (7.33-8.55 months), p<0.001; PFS: 64 3.92-5.35 months *vs.* 4 9.5 (8.1-10.91) months, p<0.001] (**Figures 1A, B**). Log-rank test of OS and PFS of 39 cases in ICIs+BCP group and 33 cases in BCP group showed that the prognosis in ICIs_BCP group was significantly better than that in BCP group [OS: 16.97 (15.11-18.84) months *vs.* 11.39 (9.70-13.08) months, P<0.05; PFS: 9.5 months, (8.1-10.9) months 6.48 (5.36-7.60) months, P<0.05] (**Figures 1C, D**).

**Table 2:** Overall response to treatment.

Best overall response	ICIs+BCP No.	ICIs No.	BCP No.
Overall	39	51	33
Complete response	0	0	0
Partial response	6	10	4
Stable disease	30	39	26
Progressive disease	3	2	3
Objective Response Rate (%)	15.38%	19.61%	12.12%
Disease Control Rate (%)	92.31%	96.08%	90.91%

### Analysis of prognostic factors

**Figure 2:**
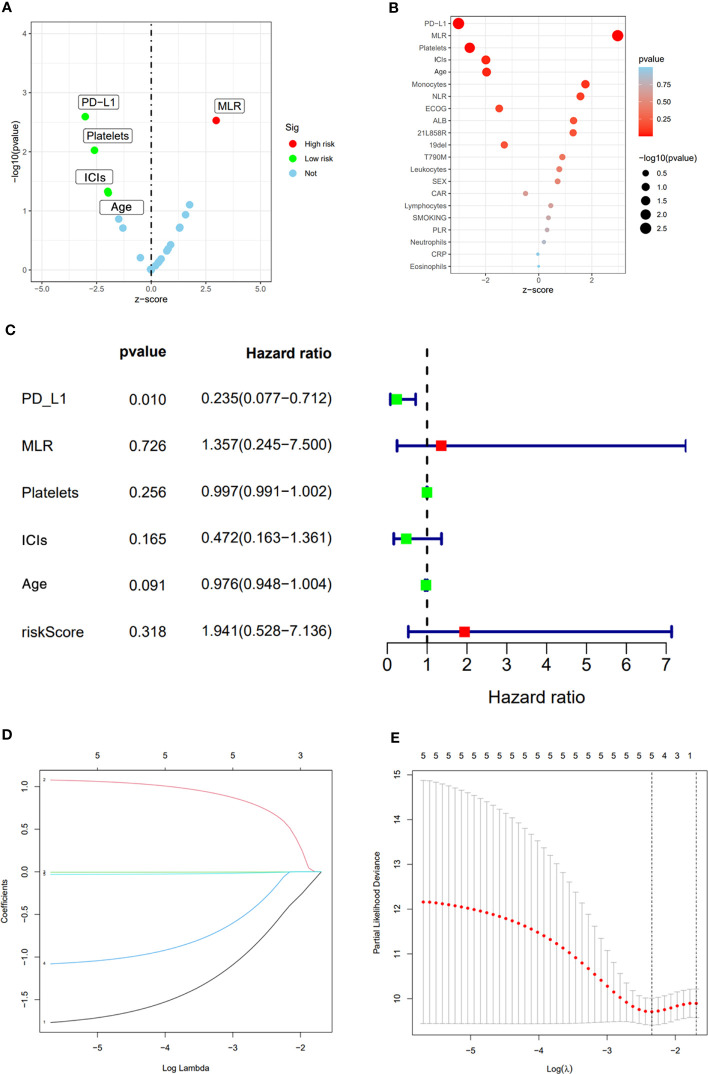
Univariate analysis results **(A, B)**. Multivariate analysis results **(C)**. LASSO Cox regression model construction, Processes of LASSO Cox model fitting **(D)**. λ selection by 10-fold cross-validation **(E)**.

After the occurrence of resistance to first-line EGFR-TKIs in the 123 NSCLC patients, univariate analysis was performed of their age, gender, smoking history, whether or not receiving immunotherapy, driver mutation type, ECOG score, PD-L1 TPS expression, neutrophil count (NEUT), lymphocyte count (LYM), monocyte count (MON), platelet count (PLT) and inflammation-related factors in serum, and neutrophil-lymphocyte ratio (NLR), monocyte/lymphocyte ratio (MLR), and platelet to lymphocyte ratio (PLR). Factors with P<0,05 in univariate analysis were subjected to multivariate analysis. the result of univariate analysis showed that PD-L1 TPS expression, MLR, PLT, whether receiving immunotherapy, and age were significant prognostic factors affecting OS in NSCLC patients after receiving first-line EGFR-TKIs therapy resistance (p<0.05) (**Table 3**), while gender, smoking history, EGFR driver mutation type, ECOG score, NEUT, LYM, MON, NLR, and PLR had no significant effect on OS of the patients. Among them, the difference between PD-L1 TPS ≥1% and PD-L1 negative patients was statistically significant (HR=0.349, 0.176-0.691, p=0.003); treatment with ICIs after drug resistance had a more significant effect on patient survival (HR=0.533, 0.286-0.991, p=0.047); higher MLR and higher EGFR-TKIs-resistance indicated a worse prognosis (HR=2.66, 1.396-5.070, p=0.003) (**Figures 2A, B**)

**Table 3:** Univariate and multivariate Cox regression analyses.

Characteristics	Univariable Analysis			Multivariate Analysis		
	HR	95% CI	*P*	HR	95% CI	*P*
PD-L1	0.349	0.176-0.691	0.003	0.235	0.077-0.712	0.010
MLR	2.660	1.396-5.070	0.003	1.357	0.245-7.500	0.726
Platelets	0.994	0.990-0.999	0.009	0.997	0.991-1.002	0.256
ICIs	0.533	0.286-0.991	0.047	0.472	0.163-1.361	0.165
Age	0.978	0.956-1.000	0.050	0.976	0.948-1.004	0.091
Monocytes	1.303	0.970-1.750	0.079	–	–	–
NLR	1.131	0.970-1.318	0.116	–	–	–
ECOG	0.597	0.302-1.179	0.137	–	–	–
ALB	1.050	0.976-1.129	0.190	–	–	–
21L858R	1.468	0.821-2.623	0.195	–	–	–
19del	0.681	0.381-1.218	0.195	–	–	–
T790M	1.415	0.658-3.042	0.374	–	–	–
Leukocytes	1.032	0.953-1.117	0.439	–	–	–
Gender	1.229	0.697-2.169	0.476	–	–	–
CAR	0.846	0.436-1.640	0.620	–	–	–
Lymphocytes	1.023	0.926-1.131	0.650	–	–	–
SMOKING	1.127	0.594-2.139	0.714	–	–	–
PLR	1.001	0.997-1.004	0.752	–	–	–
Neutrophils	1.019	0.846-1.227	0.844	–	–	–
CRP	1.000	0.976-1.024	0.975	–	–	–
Eosinophils	0.999	0.283-3.524	0.998	–	–	–

The significant prognostic factors in univariate analysis were subjected to multifactorial COX regression analysis, and the result showed that PD-L1 TPS expression was an independent prognostic factor (HR=0.235, 0.077-0.712, p=0.01), while MLR (HR=1.357, 0.245-7.500, p=0.726), PLT (HR=0.997 0.991-1.002, p=0.256), whether receiving immunotherapy (HR=0.472, 0.163-1.361, p=0.165), and age (HR=0.976, 0.948-1.004, p=0.091) were not statistically significant (**Figure 2C**).

LASSO Cox regression includes a total of 21 variables including age, gender, smoking history, whether or not receiving immunotherapy, driver mutation type, ECOG score, PD-L1 TPS expression, NEUT, LYM, MON, PLT, NLR, MLR, and PLR. 5-fold cross-validation in our study showed PD-L1 TPS expression, MLR, PLT, whether or not receiving immunotherapy and age remained the five non-zero coefficient variables as OS significant predictors (**Figures 2D, E**).

### Nomogram of the prediction model

**Figure 3:**
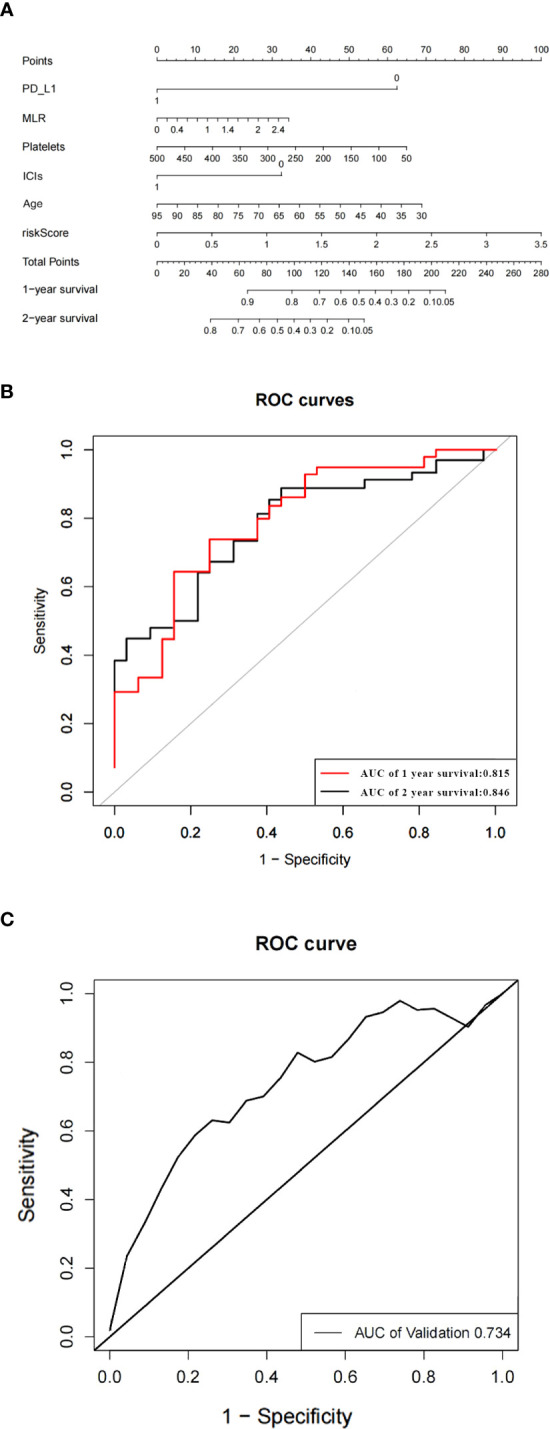
The nomogram of the overall survival prediction model **(A)**. The area under the ROC curve (AUC) indicates that the prediction model has good prediction accuracy **(B)**. ROC curve of predictive model from validation set **(C)**.

Based on the predictors obtained from the above univariate and multivariate analyses, a prediction model for the probability of patient survival after EGFR-TKIs resistance was constructed. The column line graph prediction model of the probability of survival of patients after EGFR-TKIs resistance was established using R software (**Figure 3A**). According to the obtained prediction model, each factor could obtain the corresponding score, and the total score was obtained by summing the corresponding scores of each factor, and the total score was projected onto the bottom probability value axis, which could predict the relative survival probability. The differentiation of the constructed Nomogram prediction model was evaluated by plotting the Receiver Operating Characteristic (ROC) based on the Nomogram prediction model and using the magnitude of the Area Under Curve (AUC) of the ROC curve. The AUC value of the EGFR and prediction model for 1- and 2-year survival after TKIs resistance were 0.815 and 0.846, respectively, which showed that the model had a good prediction effect and did not show significant overfitting (**Figure 3B**). We established an external validation curve using a dataset that consisted of 20 NSCLC patients meeting inclusion criteria from February 1, 2022 to January 31, 2023 to validate the predictive power of the nomogram (**Figure 3C**). The AUC value was 0.734.

## Discussion

Several previous studies have demonstrated the poor efficacy of PD-1/L1 inhibitors in patients resistant to epithelial growth factor receptor- tyrosine-kinase inhibitors (EGFR-TKIs). A KEYNOTE-001 phase II trial reported that 11 of the 25 patients with positive EGFR mutations treated with pembrolizumab monotherapy discontinued the treatment because of failure to respond to the treatment (7). A Checkmate 012 trial used Nivolumab monotherapy in EGFR mutation-positive patients, with unsatisfactory outcomes (ORR=14%; mPFS=1.8 months) (8), suggesting an unclear role of immunotherapy in patients resistant to EGFR-TKIs. Several previous studies have demonstrated that high PD-L1 expression, high TMB expression, and high CD8+ T cell infiltration often suggest good immunotherapy efficacy, especially in NCSCLC patients with high PD-L1 expression. A phase 3 Checkmate 057 clinical trial randomized 582 patients with lung adenocarcinoma who failed to respond to first-line chemotherapy into a group receiving docetaxel second-line chemotherapy and a group receiving a second-line chemotherapy. The result of their subgroup analysis based on PD-L1 expression levels (≥1%, ≥5% and ≥10%) showed that the Nivolumab monotherapy group was superior to the docetaxel second-line chemotherapy group in patients with positive PD-L1 expression (9).The level of PD-L1 expression remains unclear in NSCLC patients resistant to EGFR-TKIs therapy. Le et al. (10) showed that the PD-L1 expression, TMB level and CD8+ T cell infiltration were all low in EGFR mutation positive patients with an immune inert phenotype in tumor cells, although this trial demonstrated *in vitro* that cells expressing EGFR mutations could significantly suppress immune cell activity, but the exact mechanism remains unclear. Some studies found that when PD-1/L1 immune checkpoint inhibitors were applied to patients with EGFR mutations, some patients showed a robust immune response, while others did not. Kohsuke et al. (11) retrospectively collected 138 EGFR mutation-positive patients who were tested again for PD-L1 expression levels after resistance to EGFR-TKIs. Paired analysis of the pre- and post-progression samples showed a significant increase in PD-L1 expression in tumor samples after EGFRTKI treatment resistance, especially for T790M-negative patients, but they were unsure whether increased PD-L1 expression could provide a survival benefit for patients resistant to EGFR-TKIs treatment.

Several previous studies such as KEYNOTE-010, ATLANTIC, and POPLAR reported their uncertainty about whether ICIs alone could achieve a survival benefit in EGFR mutation-positive patients, because they found that the efficacy of ICIs was not superior to that of conventional platinum-containing two-drug chemotherapy (12–14). ICIs combined with platinum-containing two-drug chemotherapy also failed to achieve survival benefit in patients resistant to EGFR-TKIs (15, 16). In contrast to immune monotherapy, immune combination with platinum-containing dual-agent chemotherapy and anti-angiogenic drug treatment strategies have yielded good results. Related studies have shown the immunomodulatory effects of vascular endothelial growth factor (VEGF) inhibitors, a highly specific pro-vascular endothelial cell growth factor, and the key role of VEGF in suppressing anti-tumor immune responses, in addition to its angiogenic effects by negatively affecting antigen-presenting cells (APCs) and effector T cells on the one hand, and enhancing the action of immunosuppressive cells such as regulatory T cells (Treg) and myeloid-derived suppressor cells (MDSCs) on the other, which in turn bind to their receptor VEGFR2 to inhibit the differentiation of monocytes to dendritic cells (DCs) and drive immune evasion by reducing DC maturation and antigen presentation. Anti-angiogenic drugs, on the other hand, reverse VEGF-mediated immunosuppression by enhancing the killing capacity of cancer cells by T-cell-mediated checkpoint inhibitors and re-sensitizing this subset of tumors to PD-L1 inhibitors (17, 18).

Several studies have demonstrated that the combination of PD-1/L1 inhibitors, platinum-containing dual-agent chemotherapy and VEGF inhibitors can improve the survival prognosis of patients with EGFR mutation-positive disease. The CT 18 study was designed to explore the efficacy, safety and predictive biomarkers of toripalimab in combination with chemotherapy as second-line therapy for patients with EGFR-mutated advanced NSCLC. The results showed that the use of toripalimab in combination with platinum-containing two-agent chemotherapy in T790M-negative patients after resistance to EGFR-TKIs resulted in an 50% ORR, median PFS of 7 months, and median OS of 23.5 months, which were all better than controls (19, 20). The ORIENT-31 study was the first phase III, double-blind, randomized, controlled study in EGFR-resistant patients, which included 444 patients with nonsquamous, NSCLC with metastatic EGFR. All of them progressed after receiving targeted therapy. Patients were randomized to a four-drug combination group (sintilimab + VEGF inhibitor + pemetrexed + cisplatin), a three-drug combination group (sintilimab + pemetrexed + cisplatin), and a two-drug combination group (pemetrexed + cisplatin), and the results of the first interim analysis showed that the four-drug combination group was superior to the two-drug group (mPFS 6.9m vs. 4.3m, HR=0.46, P<0.0001) (21). The IMpower150 study is a phase III clinical trial exploring atezolizumab in combination with bevacizumab and carboplatin and paclitaxel (ABCP) in the first-line treatment of patients with advanced NSCLC. In patients with EGFR mutations, the efficacy in ABCP group was better than that in bevacizumab combined with carboplatin and paclitaxel group (mOS 29.4m *vs.* 18.1m, HR=0.6, 95% CI:0.31-1.14) (22). In the present study, we retrospectively analyzed 123 NSCLC patients who were previously EGFR mutation positive and resistant to treatment with EGFR-TKIs, the median PFS in the immune four-drug combination group was better than that in the other two treatment regimen groups, which is consistent with the experimental result of the ORIENT-31 study. In addition, the NCT03647956 trial also included patients with EGFR-mutated NSCLC who progressed after treatment with EGFR-TKIs. In patients who received a combination of atezolizumab (1200 mg), bevacizumab (7.5 mg/kg), pemetrexed (500 mg/m2) and carboplatin (AUC 5) every 3 weeks, with maintenance treatment with atezolizumab + bevacizumab + pemetrexed after 6 cycles, the ORR was 62.5%, the median PFS was 9.4 months (95% CI: 7.6-12.1), and the 1-year OS rate was 72.5% (95% CI: 0.56-0.83). in addition, PFS was significantly improved with the four-drug combination regimen compared with PFS with EGFR-TKIs-containing regimen rechallenge (5.8 months [95% CI 3.9-10.0 months]) and PFS with EGFR-TKIs single-drug rechallenge treatment (4.0 months [95% CI: 1.3-4.6 months]).

In this study, we enrolled 123 patients with NSCLC who were resistant to first-line EGFR-TKIs and analyzed the clinical efficacy of PD-1/PD-L1 inhibitors by counting PFS, OS, ORR and DCR of all patients to explore the efficacy of ICIs as second-line treatment in patients with EGFR-TKIs-resistant advanced NSCLC. The results showed that for patients with advanced NSCLC after resistance to EGFR-TKIs, PD-1/L1 immune checkpoint inhibitors combined with bevacizumab in combination with platinum-containing two-drug chemotherapy had some efficacy in terms of patient survival and toxicity tolerance as compared with conventional platinum-containing two-drug chemotherapy.

## Conclusions

Our study demonstrated that the PD-1/L1 immune checkpoint inhibitors combined with bevacizumab in combination with platinum-containing two-drug chemotherapy were effective in patients with advanced NSCLC after resistance to EGFR-TKIs, in whom survival was better than that in patients receiving conventional platinum-containing two-drug chemotherapy. Combination of patients’ PD-L1 TPS expression, MLR, PLT, whether or not receiving immunotherapy, age and other clinical indicators were used for survival prediction of patients with resistance to EGFR-TKIs, which enables better individualized treatment and prognosis assessment.

## Data availability statement

The original contributions presented in the study are included in the article/supplementary material. Further inquiries can be directed to the corresponding author.

## Ethics statement

All procedures performed in studies involving human participants were in accordance with the ethical standards of the institutional and/or national research committee and with the 1964 Helsinki Declaration and its later amendments or comparable ethical standards. The study was approved by the institutional review boards of all participating institutions (Approval No. 2021SL018).

## Author contributions

KW and HT designed the research study. KW, CZ, YC, and XF collected cases. KW analyzed and interpreted patient data. KW and CZ wrote the manuscript. All authors have read and approved the final manuscript. All authors contributed to the article and approved the submitted version.
